# The Anti-Infectious Role of Sphingosine in Microbial Diseases

**DOI:** 10.3390/cells10051105

**Published:** 2021-05-04

**Authors:** Yuqing Wu, Yongjie Liu, Erich Gulbins, Heike Grassmé

**Affiliations:** 1 Department of Molecular Biology, University of Duisburg-Essen, Hufelandstrasse 55, 45122 Essen, Germany; Yuqing.Wu@uk-essen.de (Y.W.); Yong-Jie.Liu@uk-essen.de (Y.L.); erich.gulbins@uni-due.de (E.G.); 2 Department of Thoracic Transplantation, Thoracic and Cardiovascular Surgery, University of Duisburg-Essen, Hufelandstrasse 55, 45122 Essen, Germany; 3 Department of Surgery, University of Cincinnati, 231 Albert Sabin Way, Cincinnati, OH 45267, USA

**Keywords:** sphingosine, sphingolipids, ceramide, sphingosine-1-phosphate, sphingosine kinases, infection, bacteria, viruses, fungi

## Abstract

Sphingolipids are important structural membrane components and, together with cholesterol, are often organized in lipid rafts, where they act as signaling molecules in many cellular functions. They play crucial roles in regulating pathobiological processes, such as cancer, inflammation, and infectious diseases. The bioactive metabolites ceramide, sphingosine-1-phosphate, and sphingosine have been shown to be involved in the pathogenesis of several microbes. In contrast to ceramide, which often promotes bacterial and viral infections (for instance, by mediating adhesion and internalization), sphingosine, which is released from ceramide by the activity of ceramidases, kills many bacterial, viral, and fungal pathogens. In particular, sphingosine is an important natural component of the defense against bacterial pathogens in the respiratory tract. Pathologically reduced sphingosine levels in cystic fibrosis airway epithelial cells are normalized by inhalation of sphingosine, and coating plastic implants with sphingosine prevents bacterial infections. Pretreatment of cells with exogenous sphingosine also prevents the viral spike protein of severe acute respiratory syndrome coronavirus-2 (SARS-CoV-2) from interacting with host cell receptors and inhibits the propagation of herpes simplex virus type 1 (HSV-1) in macrophages. Recent examinations reveal that the bactericidal effect of sphingosine might be due to bacterial membrane permeabilization and the subsequent death of the bacteria.

## 1. Introduction

**Figure 1:**
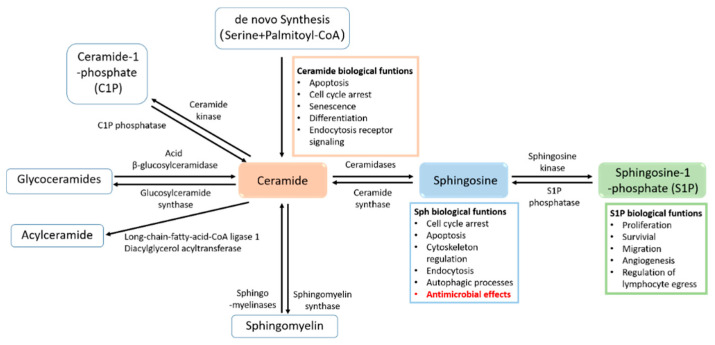
Sphingolipid metabolism and their biological functions. There are several pathways to synthesize ceramide. The *de novo* pathway generates ceramide after initiation with serine and palmitoyl CoA. Ceramides are then converted into other complex sphingolipids, including sphingomyelin, glycoceramides, and ceramide-1-phosphate. Ceramides can be acylated to acylceramide or deacylated by ceramidase to sphingosine. Sphingosine kinases phosphorylate sphingosine to sphingosine-1-phosphate. In turn, sphingolipid catabolic pathways result in ceramide from sphingomyelin, ceramide-1-phosphate, glycosphingolipids, and sphingosine. Ceramide, sphingosine, and sphingosine-1-phosphate are biologically active sphingolipids that are involved in several cellular processes.

**Figure 2:**
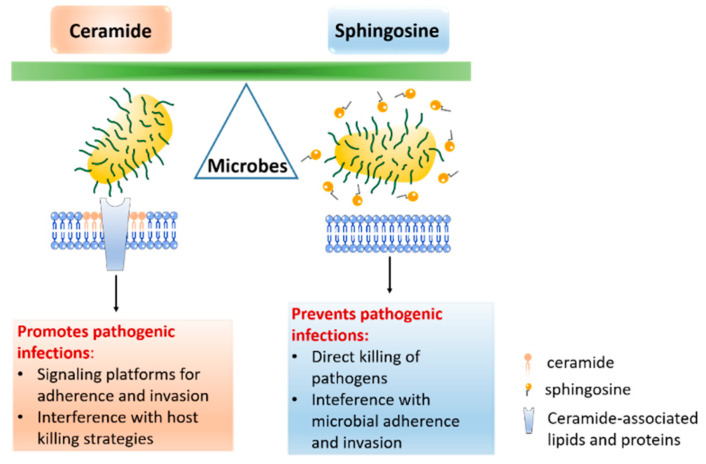
Model illustrating the different effects of ceramide and sphingosine on microbial infections. While sphingosine prevents many bacterial, viral and fungal infections, ceramide often promotes pathogenic infections, by mediating adhesion and internalization or by interfering with the killing strategies of the host.

Sphingolipids are a class of inter-convertible bioactive lipids that have dynamic functions in cellular signaling and membrane composition. All sphingolipids consist of a hydrophobic sphingoid skeleton, which contains a hydrocarbon chain, an amine group, and two hydroxyl groups. The amine group is bound to a fatty acid that, depending on the sphingolipid, has a different chain length and degree of saturation. One of the two hydroxyl groups can be changed to a phosphate, phosphocholine, or carbohydrate [1]. It has long been understood that sphingolipids serve as structural components of cell membranes, but multiple recent studies have also described their crucial functions in the regulation of physiological and pathological processes [2,3,4,5]. The key molecules of sphingolipid signaling are ceramide, sphingosine, and sphingosine-1-phosphate (S1P), which are involved in very diverse cell processes, such as proliferation, endocytosis, necrosis, apoptosis, and migration [2,5,6,7] (Figure 1). Sphingolipids are also important membrane components for pathogens, which use these components as receptors to adhere to the host cell membrane. In addition, sphingolipids, particularly sphingomyelin and ceramide, together with cholesterol lipid rafts, act as signaling platforms for adherence and invasion receptors [8] (Figure 2). This review focuses on sphingosine and its role in infectious diseases, and briefly discusses ceramide—a bioactive sphingolipid—and its derivative, sphingosine-1-phosphate.

Ceramide is formed either by the hydrolysis of sphingomyelin through the activity of neutral, acidic, or alkaline sphingomyelinases or through *de novo* synthesis and/or the breakdown of complex sphingolipids. Several bacteria, viruses, and parasites exploit acid sphingomyelinase and/or the neutral sphingomyelinase–ceramide system to infect mammalian cells (for a recent review, see [9]). Ceramide enters a metabolic pathway and can be converted into sphingosine by the action of (acid) ceramidase. Sphingosine is further metabolized by phosphorylation via an ATP-dependent sphingosine kinase (Sphk), which leads to sphingosine-1-phosphate (S1P) [10].

Sphingosine 1-phosphate (S1P) regulates the proliferation, survival, and migration of mammalian cells through both extracellular receptor-mediated and intracellular mechanisms, either through intracellular targets or by activating a family of specific G-coupled receptors (S1PR) [11]. S1P is produced during inflammation and upon tissue damage, and it has well-described roles in cell signaling, the cell death/survival decision, and mediation of the pro-inflammatory response, including in the context of immunity [12]. An increasing number of reports describe the ability of pathogens, including mycobacteria, different viruses, and parasites, to dysregulate S1P signaling by modulating the Sphk/S1P axis reviewed in [13]. Therefore, agents targeting the generation of S1P are being actively developed as therapeutics for cancer and inflammatory and infectious diseases.

Sphingosine (Sph) constitutes a class of natural products containing a long aliphatic chain with a polar 2-amino-1,3-diol terminus (2-amino-4-trans-octadecene-1,3-diol). It occurs in the cell membranes of all animals and many plants and plays an important role in various complex biological processes, such as cell growth, differentiation, autophagic processes and development [14,15]. After its release from complex sphingolipids, sphingosine is mainly reacylated by ceramide synthase or phosphorylated by sphingosine kinase to generate sphingosine-1-phosphate [16] (Figure 1). Sphingosine has been connected with a variety of cellular processes, such as the induction of cell cycle arrest and apoptosis by modulating protein kinases and other signaling pathways [14]. It has roles in regulating the actin cytoskeleton and endocytosis, and has been shown to inhibit phosphokinase C (PKC) [17]. In addition to its functions in cell signaling, sphingosine has broad-spectrum antimicrobial properties. The antimicrobial activity of sphingosine has been described for Gram-positive and Gram-negative bacteria [18], enveloped viruses [19], and fungi [20] (Table 1). Sphingosine’s role as an antimicrobial is important in tissues such as the skin, respiratory epithelium, and the oral cavity. Chronic diseases, such as cystic fibrosis (CF), in which the normal sphingosine level is reduced in epithelial cells [21,22], are associated with problems related to high infection susceptibility. Therefore, exogenous sphingosine may be a successful antimicrobial therapeutic. Inhaled nebulized sphingosine has been shown to be effective in both preventing and treating pneumonia in multiple CF mouse models without producing severe toxic side effects [21,22,23,24].

## 2. The Role of Sphingosine in Infectious Diseases

### 2.1. Sphingosine and Bacteria

Since the middle of the last century, microbiologists have recognized that the skin is a natural barrier in the defense against microbial infections, and that skin lipids can reduce infections caused by Gram-positive bacteria [33,38]. However, due to methodological limitations and historical reasons, these lipids were not completely identified until the end of the 20th century. Since then, several reports on the broad-spectrum antimicrobial properties of sphingosine have been published. Sphingosine has been shown to have remarkably potent antibacterial activity against a variety of pathogens, including *Pseudomonas aeruginosa, Staphylococcus aureus, Acinetobacter baumannii, Haemophilus influenzae, Burkholderia cepacia, Moraxella catarrhalis, Escherichia coli, Fusobacterium nucleatum, Streptococcus sanguinis, Streptococcus mitis, Corynebacterium bovis, Corynebacterium striatum*, and *Corynebacterium jeikeium* [20,21,23,26,33,39,40]. Among these pathogens, *Staphylococcus aureus* and *Pseudomonas aeruginosa* are the most studied. Therefore, herein, we focus on the bactericidal effect of sphingosine, as well as the mechanisms behind it, on these two bacterial species.

#### 2.1.1. *Staphylococcus aureus*

*Staphylococcus aureus* (*S. aureus*) is a ubiquitous and opportunistic Gram-positive coccoid bacterium, and a prevalent skin pathogen. It causes severe respiratory tract and systemic infections, especially among patients with previous viral infections, burn wounds, trauma, or sepsis; those requiring mechanical ventilation; and those with cystic fibrosis (CF) (for recent reviews, see [41,42]). Treating *S. aureus* infections is an increasing challenge due to multi-drug resistance against common antibiotics caused by methicillin-resistant *Staphylococcus aureus* (MRSA).

Bibel et al. first described the antimicrobial activity of the skin lipid sphingosine in 1992 [25]. The authors found that sphingosines were profoundly effective against *S. aureus* (strain 502A), with a 4-log reduction at 20 µM and a 2-log reduction at 2.5 µM. The optimal inhibition was observed after 60 min incubation at 37 °C and pH 6.5, and the antimicrobial activity of sphingosines was Ca^++^ dependent. Accordingly, Arikawa et al. found a significantly downregulated sphingosine level in patients with atopic dermatitis, which was caused by the decreased activity of acid ceramidase [39]. The decreased sphingosine level was associated with enhanced vulnerability to colonization by *S. aureus* in atopic dermatitis patients. Parsons et al. further reported that toxic fatty acids, including sphingosine, permeabilized the membrane and released the pathogen’s intracellular ATP into the medium without causing complete dissolution of the cytoplasmic membrane or a significant morphological change [34]. Other groups observed that sphingosine caused multiple cell wall lesions, membrane evaginations, the loss of ribosomes, and ultrastructural damage in *S. aureus* through the use of electron microscopy [26,33].

In addition to skin infections, *S. aureus* infections often occur in chronic obstructive pulmonary disease (COPD) and cystic fibrosis (CF) patients [43,44,45,46]. Cystic fibrosis is a disease caused by mutations in the cystic fibrosis transmembrane conductance regulator (CFTR) protein, and it is the most widespread recessively inherited disorder in North America and Europe [46,47,48,49]. Notably, the most common cause of morbidity and mortality in CF patients is chronic pulmonary infection with bacterial pathogens, particularly *Pseudomonas aeruginosa* (*P. aeruginosa*) and *S. aureus*. [50]. CF mice showed a marked sphingosine reduction and ceramide accumulation in the respiratory tract due to the repressed activity of acid ceramidase [21,45,51]. Following the discovery of low levels of sphingosine in CF mice [21], Tavakoli et al. studied the bactericidal effect of sphingosine and ceramide on *S. aureus* (strain E25) in CF mice [23]. The authors confirmed that CF mice had a high susceptibility to *S. aureus* infection compared with wild-type mice. More importantly, inhalation of C18-sphingosine 30–40 min before infection could protect the CF mice from infection with *S. aureus*. This work was the first to report that sphingosine administered by inhalation acted against *S. aureus* in airways and could prevent airway infection. Based on these findings, the use of sphingosine on implanted medical devices was proposed to prevent staphylococcal infections [29,35]. About 25% of mechanically ventilated patients are affected by ventilator-associated pneumonia, which has an estimated mortality of 13% [52]. Both Gram-negative and Gram-positive bacteria cause ventilator-associated pneumonia, and *S. aureus* is the most common Gram-positive bacterium responsible for this disease [53] (we later discuss Gram-negative bacteria, specifically *P. aeruginosa*). Seitz et al. coated the plastic surfaces of endotracheal tubes with high sphingosine concentrations. As a result, the adherence and growth of methicillin-resistant *S. aureus* (MRSA) on the plastic surface were significantly reduced [29].

Along with *S. aureus*, *Staphylococcus epidermidis* (*S. epidermidis*) is a dominant skin colonizer and is prevalent in orthopedic infections. The inhibitory effect of sphingosine (10 µM) on *S. epidermidis* was described by Beck et al. [35], who expanded the application of sphingosine to implant materials [35]. Periprosthetic infection is a devastating complication of joint replacement surgery, and results in bacterial biofilm formation [54]. Bacteria that adhere to implant surfaces produce a complex hydrated matrix of glycocalyx that coats the bacteria and forms a biofilm layer [55]. The authors generated *S. epidermidis* biofilms on different implant materials and determined the bactericidal effect of sphingosine on the formed biofilms. When a concentration of 100 µM was used for coating, sphingosine eliminated at least 94% of the pathogens. Coating implant samples (titanium, steel, and polymethylmethacrylate) with sphingosine prevented implant contamination and resulted in a significant reduction in biofilm formation on the implant surfaces [35].

#### 2.1.2. *Pseudomonas aeruginosa*

*Pseudomonas aeruginosa* (*P. aeruginosa*) is an opportunistic Gram-negative rod-shaped bacterium that, similar to *S. aureus*, is quite often associated with burn wounds, trauma, sepsis, cystic fibrosis, and chronic obstructive pulmonary disorders (COPD), and it causes significant infections in patients who need mechanical ventilation [43,44,56,57,58]. Worldwide, *P. aeruginosa* is known to be one of the most common Gram-negative pathogens associated with pneumonia [59] and is, in addition to *S. aureus,* the leading pathogen and main cause of death in CF patients [56,57,58,60,61,62].

The high susceptibility of cystic fibrosis mice and patients to *P. aeruginosa* infections was found to be significantly reduced by treatment with sphingosine, which is extensively downregulated in the tracheal and bronchial epithelia of cystic fibrosis patients and mice [21]. The inhalation of sphingosine restores the reduced sphingosine levels of CF tracheal and bronchial epithelia, thereby protecting mice from infection with *P. aeruginosa* (strain PA14, ATCC 27853, and ATCC 762).

In 2017, our group showed that the high susceptibility of cystic fibrosis patients and mice to *P. aeruginosa*, *S. aureus,* and *Acinetobacter baumanii* was related to β1-integrin accumulation due to the increased ceramide level on the luminal membrane upper-airway epithelial cells in these hosts [22]. The results revealed that the accumulation of ceramide in cystic fibrosis cells trapped β1-integrins in the luminal membrane of CF bronchial, tracheal, and nasal epithelial cells. Ectopic β1-integrins in the luminal membrane downregulate the expression of acid ceramidase (which converts ceramide to sphingosine) in human and murine CF airway epithelial cells. Reduced acid ceramidase expression further mediates ceramide accumulation, thereby forming a positive feedback loop between ceramide and β1-integrins with the concomitant depletion of sphingosine. The blockade of β1-integrin and this vicious cycle normalizes sphingosine levels in epithelial cells from cystic fibrosis patients and cystic fibrosis mice and prevents them from developing severe *P. aeruginosa* infections. This work explained the low level of sphingosine in the cystic fibrosis model [22]. It remains unknown how β1-integrin manipulates acid ceramidase and how CFTR deficiency leads to the β1-integrin-mediated downregulation of acid ceramidase. Following the finding that sphingosine prevents infections in the cystic fibrosis model, sphingosine was also proven to prevent *P. aeruginosa* infections in other mice models, such as ceramidase synthase-2-deficient, aged, burn-injured, and septic mice [21,30,31,32]. Interestingly, the genetic deletion of sphingosine kinase (Sphk), which phosphorylates the lipid sphingosine to generate sphingosine-1-phosphate (S1P), also protected mice from *P. aeruginosa*-mediated lung inflammation [63,64], further supporting an anti-bacterial effect of sphingosine, which very likely accumulated upon deletion of the kinase.

Fischer et al. investigated the uptake of several sphingoid bases, including sphingosine, by *Escherichia coli* (*E. coli*) and *S. aureus* and assessed their subsequent ultrastructural changes via electron microscopy. While sphingosine-treated *S. aureus* underwent drastic membrane disruption, the cytoplasmic and outer membranes of *E. coli* appeared to remain intact. Both *E. coli* and *S. aureus* cells contained unique internal inclusion bodies that may reflect lipid uptake [26]. These observations excluded the possibility that sphingosine kills bacteria by simple lysis or destruction of the pathogen structure. Recent findings from our group shed light on the mechanism of sphingosine-mediated bacterial killing [27]. The results indicate that sphingosine has an *in vitro* bactericidal effect on *P. aeruginosa* and *S. aureus*. Upon treatment with sphingosine, the permeabilization of the bacterial membranes, the release of intracellular ATP, decreased metabolic activity, and reduced bacterial survival were observed. This bactericidal effect depends on the NH_2_ group of sphingosine. NH_2_ groups can be protonated and are positively charged under neutral and slightly acidic pH [65]. This might explain the observation that sphingosine maintains its bactericidal activity only under neutral or acidic pH, whereas this effect is profoundly reduced at alkaline pH. Positively charged sphingosine NH_2_ groups interact with cardiolipin in pathogens and effectively kill bacteria, while bacteria that lack cardiolipin synthase are resistant to sphingosine treatment. Incorporating cardiolipin into membranes results in a negative curvature [66]. The killing mechanism mediated by the interaction between sphingosine and cardiolipin might be the following: when negatively charged cardiolipin binds to sphingosine, cardiolipin may aggregate and form rigid, gel-like membrane domains. These domains disturb the original membrane structure and lead to membrane permeabilization and bacterial death. This theory should be further verified.

#### 2.1.3. *Neisseria gonorrhoeae*

*Neisseria gonorrhoeae (N. gonorrhoeae*) is a Gram-negative diplococcus and an obligate human pathogen. Gonococci are the causative agents of the sexually transmitted disease gonorrhea and have the potential to enter the bloodstream and cause systemic disseminated infections with severe consequences, such as endocarditis and arthritis [28,67,68]. An increasing number of reports are highlighting the threatening development of multidrug-resistant gonococci; thus, new strategies are required to combat this pathogen. Solger et al. showed that *N. gonorrhoeae* was also sensitive to sphingosine treatment [68]. Treatment with 20 µM sphingosine achieved a similar bacterial killing effect to the antibiotic kanamycin [68]. Mechanistically, it was shown that invasive *N. gonorrhoeae* strains ingest sphingosine from the host cell, which is evenly distributed on the surface of intracellular bacteria, and then incorporates it into the bacterial membrane. This leads to the decreased survival of *N. gonorrhoeae* and confirms that sphingosine can directly affect bacteria rather than activating other cellular bactericidal factors. However, whether sphingosine kills *N. gonorrhoeae* by interacting with cardiolipin, as in *P. aeruginosa*, is unknown and should be studied accordingly.

### 2.2. Sphingosine and Viruses

Viruses are obligate intracellular pathogens: they manipulate the host and exploit membranes and their components, such as sphingolipids, in all steps of their life cycle, including adhesion and membrane fusion, viral replication, and budding from host cells [69,70,71]. Human immunodeficiency virus (HIV), measles virus (MV), Ebola virus (EBOV), Sindbis virus (SINV), and rhinovirus [72,73,74,75] all exhibit sphingolipid-dependent virus entry by activating sphingomyelinases and making use of ceramides as adherence and signaling platforms.

Sakamoto et al. were the first to show that targeting hepatitis C virus (HCV) sphingolipid metabolism could inhibit viral replication and prevent infection [19]. They identified a new lipophilic long-chain base compound, NA255, which has a sphingolipid-binding motif that can directly interact with sphingomyelin. Hepatitis C virus substantially affects lipid metabolism, and the remodeling of sphingolipids appears to be essential for HCV persistence in vitro. The quantification of serum sphingolipid variations in patients with acute HCV infection revealed that sphingosine and sphinganine levels were significantly upregulated in patients who were unable to clear the virus over time compared with patients who spontaneously cleared the infection [19]. The persistence of HCV after acute infection induces the downregulation of C24-ceramide and the simultaneous elevation of serum sphingosine and sphinganine concentrations [76].

Since then, the direct effect of sphingolipids on viruses has been rarely reported, which has only recently changed [36,37]. Lang et al. first reported that sphingosine blocked the propagation of herpes simplex virus type 1 (HSV-1) in macrophages [36]. They found that the restriction of HSV-1 reproduction was dependent on cellular acid ceramidase. HSV-1 is an enveloped DNA virus that replicates in several cell types [77]. After targeting a cell, HSV-1 either fuses with the cell plasma membrane or is taken up by the cell into endosomes, where fusion with the perimeter of membrane leads to infection [78,79,80]. Lang et al. showed that the deficiency of acid ceramidase led to the uncontrolled replication of HSV-1. In macrophages, acid ceramidase converts ceramide into sphingosine, which forms sphingosine-rich intraluminal vesicles that can bind to HSV-1 particles. This limits the fusion of HSV-1 with the endosomal membrane, prevents cellular infection, and releases the virus for lysosomal degradation. Correspondingly, macrophages treated with sphingosine blocked HSV-1 replication. This research was the first study that showed the direct antiviral effect of endogenous sphingosine [36].

**Figure 3:**
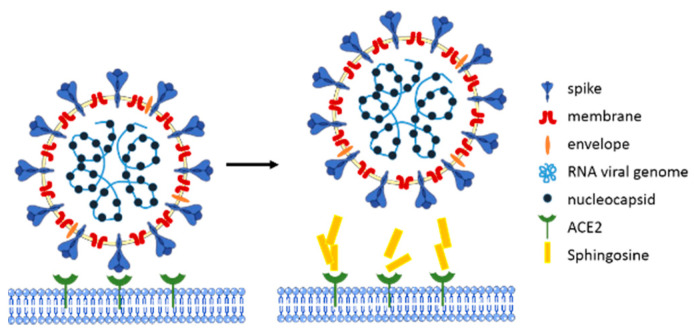
Model of how sphingosine prevents SARS-CoV-2 infection. Exogenous sphingosine application in human nasal or Vero-E6- epithelial cells results in the binding of sphingosine and angiotensin-converting enzyme 2 (ACE2) receptor, which is required for severe acute respiratory syndrome coronavirus-2 (SARS-CoV-2) infection. Sphingosine thereby prevents the recognition and interaction between the spike protein of SARS-CoV-2 and the host’s ACE2 receptor and ultimately protects the cells from infection.

Recently, some reports have been published on the role of lipid raft components in the infection of the severe acute respiratory syndrome coronavirus-2 (SARS-CoV-2) of host cells [81,82,83]. SARS-CoV-2 is the causative agent of coronavirus disease 2019 (COVID-19), which is associated with a high mortality rate [84]. SARS-CoV-2 infects cells using its viral spike glycoprotein, which interacts with human epithelial cells via its cellular angiotensin-converting enzyme-2 (ACE2) receptors [85,86,87,88]. Lipid rafts, which are membrane domains enriched with cholesterol and sphingolipids such as gangliosides, have been described as a perfect interface for the concentration of the ACE2 receptor on epithelial cell membranes, thus facilitating the interaction with the viral spike proteins [81,82,83]. Another exciting study showed that sphingosine plays a protective role against infection with SARS-CoV-2 [37]. Edwards et al. used pseudo-viral particles expressing SARS-CoV-2 spike (pp-VSV–SARS-CoV-2 spike), serving as a bona fide system that mimics SARS-CoV-2 infection [37]. The study utilized Vero-E6 epithelial cells and freshly isolated human nasal epithelial cells to examine whether exogenous sphingosine prevents pp-VSV–SARS-CoV-2 infection of epithelial cells. The authors showed that pretreating cells with exogenous sphingosine blocked the interaction between the viral spike protein and ACE2 (Figure 3). This inhibition was achieved with a relatively low concentration of sphingosine (only 0.25 µM), and it could completely block the interaction between the spike protein and ACE2 receptor at 2 µM. At this concentration, sphingosine had no side effects or toxic effects on human nasal epithelial cells. Therefore, it provides a potential defense strategy against SARS-CoV-2 infection [37]. To date, it is still unknown whether sphingosine has a direct antiviral effect on SARS-CoV-2 or HSV-1.

Recent studies indicate that fingolimod (FTY720), a sphingosine analog, already approved for the treatment of multiple sclerosis (MS), may help prevent the more serious neurological side-effects of SARS-CoV-2 infection [89,90,91]. FTY720 requires sphingosine kinase (Sphk) for activation and phosphorylation and blocks the inflammatory reaction mediated by sphingosine-1-phosphate (S1P)/sphingosine-1-phosphate receptor (S1PR)1 [92,93]. Currently, as one of the most unique sphingolipid-based drugs, fingolimod has good potential to become an important drug in the treatment of COVID-19.

### 2.3. Sphingosine and Fungi

Fungal infections are increasingly coming into focus due to the continual rise of immune-deficient patients, such as those who have HIV/AIDS or take immunosuppressant medications. Cryptococcosis, candidiasis, and aspergillosis are severe invasive mycoses with high mortality in immunocompromised patients [94]. The fungal eukaryotic cell membrane contains sphingolipids, in addition to other components [95]. Because fungal sphingolipids have different chemical structures from those of mammalian sphingolipids, they can be exploited as targets for the development of antifungal drugs. Therefore, several studies have focused on sphingolipid metabolism in fungal cells for the development of new antifungal agents [20,96]. A variety of compounds have been reported to act against the fungal sphingolipid biosynthetic pathway. Such compounds include natural and synthetic molecules, such as fumonisin B1 as an inhibitor of ceramide synthase or FTY720 (fingolimod) as a sphingosine-1-phosphate antagonist, as well as antibodies, which can attenuate fungal sphingolipids [20,96]. In recent years, monoclonal antibodies against fungal glucosylceramides (GlcCer) have been developed and proven to inhibit cryptococcal growth in vitro [97]. Infection with *Candida albicans* was shown to be sensitive to inositol phosphorylceramide synthase inhibitors [98], and glucosylceramide and galactosylceramide synthase inhibitor blocked the germination and hyphal growth of *Aspergillus nidulans* [99]. An increasing threat is the progressive development of drug resistance to currently available antifungal drugs. Thus, there is an urgent need for antifungal compounds with novel mechanisms of action. The direct use of sphingosine as an antifungal agent was shown in 1992 when Bible et al. proved that sphingosine has antifungal activity against *Candida albicans*. Sphingosine was found to be fungistatic and could prevent germination and delay thalli formation [33]. The application of sphingosine might disrupt the balance of eukaryotic sphingolipid metabolism and affect the growth of fungi. Further reports on the utility of sphingosine as an antifungal agent are pending.

## 3. Conclusions

Sphingosine, as a lipid common to the skin and oral cavity, has remarkable antimicrobial activities against a variety of Gram-positive and Gram-negative bacteria, viruses, and fungi (Table 1). The presented review indicates that sphingosine may be useful as a coating for plastic surfaces and orthopedic implants, thereby preventing bacteria-induced wound infections, and as a medicinal treatment inhaled by the host, i.e., as a protective agent against infections in cystic fibrosis disease. These promising data provide evidence that sphingosine may be a novel antimicrobial agent that can prevent bacterial adherence and induce the killing of pathogens. Therefore, it may contribute to defensive barrier functions and has the potential for use in prophylactic or therapeutic interventions in infection. Sphingosine even seems to positively influence several significant viral infections, such as HCV and SARS-CoV-2, by interfering with the interaction between the virus and its receptor, and it might be helpful as an antifungal agent. Further preclinical and, eventually, clinical examinations of sphingosine are warranted to evaluate its potential use as a prophylactic or early treatment for microbial diseases.

## Figures and Tables

**Table 1:** Microbes attacked by sphingosine.

Microbes	Species	References
bacteria	*Acinetobacter baumannii*	[21]
	*Brevibacterium epidermidis*	[25]
	*Burkholderia cepacia*	[21]
	*Corynebacterium bovis*	[18]
	*Corynebacterium striatum*	[18]
	*Corynebacterium jeikium*	[18]
	*Escherichia coli*	[18,26,27]
	*Haemophilus influenzae*	[21]
	*Micrococcus luteus*	[25]
	*Moraxella catarrhalis*	[21]
	*Neisseria gonorrhoeae*	[28]
	*Propionibacterium acnes*	[25]
	*Pseudomonas aeruginosa*	[21,22,25,27,29,30,31,32]
	*Staphylococcus aureus*	[18,23,25,27,29,33,34]
	*Staphylococcus epidermidis*	[35]
	*Streptococcus mitis*	[18]
	*Streptococcus pyogens*	[25]
	*Streptococcus sanguinis*	[18]
viruses	*Hepatitis C virus*	[19]
	*Herpes simplex virus type 1*	[36]
	*SARS-CoV-2*	[37]
fungi	*Candida albicans*	[25]
	*Epidermatophyton floccosum*	[33]
	*Trichophyton mentagrophytes*	[33]
	*Trichophyton tonsurans*	[33]

## Data Availability

Not applicable.

## Abbreviations

Sphingosine (Sph); Sphingosine-1-phosphate (S1P); Sphingosine kinase (Sphk); *Staphylococcus aureus* (*S. aureus*); *Pseudomonas aeruginosa* (*P. aeruginosa*); cystic fibrosis (CF); cystic fibrosis transmembrane conductance regulator (CFTR) protein; *Neisseria gonorrhoeae (N. gonorrhoeae*); hepatitis C virus (HCV); herpes simplex virus type 1 (HSV-1); severe acute respiratory syndrome coronavirus-2 (SARS-CoV-2); angiotensin-converting enzyme 2 (ACE2); human immunodeficiency virus (HIV)
